# Changes in Volatile and Non-Volatile Flavor Chemicals of “Valencia” Orange Juice over the Harvest Seasons

**DOI:** 10.3390/foods5010004

**Published:** 2016-01-04

**Authors:** Jinhe Bai, Elizabeth A. Baldwin, Greg McCollum, Anne Plotto, John A. Manthey, Wilbur W. Widmer, Gary Luzio, Randall Cameron

**Affiliations:** USDA, ARS, U.S. Horticultural Research Laboratory, 2001 S. Rock Rd, Fort Pierce, FL 34945, USA; liz.baldwin@ars.usda.gov (E.A.B.); greg.mccollum@ars.usda.gov (G.M.); anne.plotto@ars.usda.gov (A.P.); john.manthey@ars.usda.gov (J.A.M.); wwidmer@gmail.com (W.W.W.); gary.luzio@ars.usda.gov (G.L.); Randall.cameron@ars.usda.gov (R.C.)

**Keywords:** *Citrus sinensis*, maturity, orange juice, volatile, aroma, flavor, bitterness, limonoid

## Abstract

Florida “Valencia” oranges have a wide harvest window, covering four months after first reaching the commercial maturity. However, the influence of harvest time on juice flavor chemicals is not well documented, with the exception of sugars and acids. Therefore, we investigated the major flavor chemicals, volatile (aroma), non-volatile (taste) and mouth feel attributes, in the two harvest seasons (March to June in 2007 and February to May in 2012). Bitter limonoid compounds, limonin and nomilin, decreased gradually. Out of a total of 94 volatiles, 32 increased, 47 peaked mid to late season, and 15 decreased. Juice insoluble solids and pectin content increased over the season; however, pectin methylesterase activity remained unchanged. Fruit harvested in the earlier months had lower flavor quality. Juice from later harvests had a higher sugar/acid ratio with less bitterness, while, many important aroma compounds occurred at the highest concentrations in the middle to late season, but occurred at lower concentrations at the end of the season. The results provide information to the orange juice processing industry for selection of optimal harvest time and for setting of precise blending strategy.

## 1. Introduction

“Valencia” is the predominant orange variety grown in Florida and is mainly used for juice. This variety is favored by the juice industry for the rich orange color and flavor [1]. The harvest season for “Valencia” oranges occurs typically from February to May or March to June, depending on the strain, year and location [2]. Florida maturity indices for oranges harvested between November 16 to July 31 are: soluble solids content (SSC, ≈ ° Brix) > 8.5%, titratable acidity (TA or acid) > 0.4%, SSC/TA ratio > 10.25, and juice content > ~ 45 mL/100 g (4.5 gal per 1.6 bushel box) [3]. There is a gradual decrease in acid by decomposition of citric acid, the principle organic acid of citrus juice, a slight increase in SSC and a consistent increase of SSC/TA ratio over the harvest season [4].

The major chemicals contributing to bitterness in orange juice (OJ) are the limonoids, limonin and nomilin. It has been noticed previously that limonin content decreased during the harvest season [5,6]. However, very little attention has been given to nomilin. Citrus greening, also known as Huanglongbing (HLB), has spread throughout the major citrus-producing regions in Florida and many other countries, and has largely accentuated OJ bitterness due to higher concentrations of limonin and nomilin in HLB-affected fruit [7,8]. This is particularly evident in “Hamlin” oranges which already have higher limonin and nomilin concentrations in healthy and HLB-affected fruit than in “Valencia” oranges [7]. Thus, since the arrival of HLB disease, bitter limonoids have become important to OJ flavor quality research.

Orange flavor is comprised of sugars, acids, limonoids, and a complex mixture of volatile compounds, of which some 200 have been identified [9]. Of those, only 20–40 are generally recognized as important aroma contributors to OJ, including esters, aldehydes, monoterpenes, sesquiterpenes, alcohols, ketones, and hydrocarbons [10,11,12,13]. Over the last half century, much effort has been focused on varietal selections, pre- and postharvest treatments, processing methods (such as extractor and finisher settings) and thermal pasteurization or concentration. Yet, little work has been published on influence of harvest maturity.

Among other factors that contribute to the quality of orange juice, the “cloud” is comprised of pectin and pulp particulates and provides some viscosity to the juice. The cloud can be destabilized or precipitated due to the action of the enzyme pectin methylesterase (PME) if this enzyme is not inactivated by heat [14]. The cloud also significantly contributes to flavor by binding hydrophobic molecules, such as volatile terpenes [15,16].

Therefore, the goal of this study was to evaluate the overall flavor quality-related chemicals of “Valencia” fruit and juice—volatile aromas, non-volatile tastes, and mouth feel attributes, over the four-month commercial harvest seasons, in order to provide information to the OJ juice processing industry for selection of optimal harvest time and for setting of precise blending strategy.

## 2. Experimental Section

### 2.1. Fruit and Juice Preparation

In the 2007 experiment, fruit were harvested from five trees in a South Florida commercial grove on March 19, April 13, May 15, and June 29. At each harvest, 30 fruit were picked from each replicate tree. After measuring peel color, the fruit were sanitized with 200 mg·L^−1^ NaOCl for 30 s, and gently hand juiced using a Sunkist J1 Commercial Citrus Orange Juicer (Sherman Oaks, CA, USA). Halved fruit were pressed onto the automatic self-reversing reamer, and seeds and segment membranes were screened by a strainer. The juice was lightly pasteurized (71 °C for 15 s in a water bath) and frozen at −20 °C until analyzed.

The 2012 experiment was similar to 2007 experiment except for the following changes: the orange grove was in the coastal Florida Indian River area, four trees were used and 20 fruit were picked from each replicate tree at each harvest. The harvest dates were 22 February, 21 March, 18 April, and 12 May. Fruit were cleaned with JBT Fruit Cleaner 395, and juiced using a fresh juicer (JBT Fresh’n Squeeze^®^ Point-of-Sale Juicer, JBT FoodTech Corporation, Lakeland, FL, USA). Unfortunately, citrus greening or Huanglongbing (HLB) disease, associated with *Liberibacter asiaticus* (*C*Las), had spread throughout the major citrus producing regions in Florida by 2010 [17], and the diseased fruit were shown to be associated with off-flavor and other negative quality defects [18,19]. Therefore, to eliminate this potentially complicating factor, the trees or fruit juice was tested for the disease before sampling. Fruit for the 2007 juice samples were harvested from trees that were *C*Las free by leaf analysis using the Li *et al.* [20] real-time quantitative polymerase chain reaction (qPCR) method. The qPCR method of Bai *et al.* [21] as modified by Zhao *et al.* [22], was used to measure *C*Las in the juice and confirm that the juice samples for the 2012 harvest were disease free.

### 2.2. General Fruit and Juice Features

Fruit weight was measured individually and juice was pooled for each replicate and quantified for juice content (mL 100·g^−1^ of fruit) calculation. Juice color was measured with a Macbeth Color-Eye 3100 spectrophotometer (Kollmorgen Instruments Corp., Newburgh, NY, USA) and expressed as color number. Insoluble solids were determined by measuring dry weight residues of pellets obtained from centrifugations of juice. Briefly, juice samples were centrifuged at 27,000× g for 30 min. Supernatants were discarded and pellets were carefully re-suspended with deionized water (equivalent amount as in original juice) and centrifuged again. The final pellets, collected after centrifugation, were vacuum dried at 55 °C. The ratio of dry weight to original juice weight represented the insoluble solids content [23].

Peel oil content was analyzed by a bromate titration method [24]. In brief, the juice sample (25 mL) and 2-propanol (25 mL) with a few boiling stones were distilled until the solvent ceased to reflux. After adding 10 mL of 4 N HCl with a drop of 0.1% methyl red indicator, peel oil content was determined by titrating the distilled fraction with 0.025 N bromide-bromate solution until the color disappeared.

### 2.3. Sugar and Acid Analyses

Juice samples, 35 mL, were centrifuged at 10,000× g for 15 min, and supernatants were used for SSC, TA and individual sugar and acid analysis. SSC was determined by using a refractometer (Atago RX-5000 cx, Tokyo, Japan). TA was determined by titrating juice supernatant to pH 8.1 with 0.1 N NaOH using an autotitrator (Metler Toledo DL50, Daigger & Company, Vernon Hills, IL, USA) and the content was calculated as citric acid on juice weight basis. Individual sugars (sucrose, glucose and fructose) were measured by HPLC [23] using a Water Sugar Pak column and refractive index detector (Perkin Elmer, Norwalk, CT, USA) and acids (citrate and malate) by HPLC equipped with an Altech OA 1000 Prevail organic acid column (Altech Corp., Flemington, NJ, USA) and a Spectra System UV 6000 LP detector (Shimadzu, Kyoto, Japan) according to Baldwin *et al.* [23].

### 2.4. Limonoid Analysis

Different extraction methods were used in 2007 and 2012. In 2007, 150 mL of juice samples were centrifuged at 10,000× g for 15 min. Supernatant (100 mL) were extracted 3 times with equal volumes of methylene chloride. Combined extracts were rotovaporated to dryness and the residues redissolved in 12 mL acetone. The solutions were filtered through a 0.45 µm PTFE filter (Siemens, Shrewbury, MA, USA), then taken to dryness with a Savant centrifugal evaporator (Milford, MA, USA). The residues were redissolved in acetone (1 mL) containing 4.35 μg hesperetin as an internal standard prior to analysis. In 2012, 2 mL of juice sample was added to 10 mL of methanol in a Teflon gasket screw-top test tube and shaken for 18 h with an orbital shaker (VSOS-4P, Pro Scientific, Oxford, UK) at 120 rpm at 25 °C. The mixtures were centrifuged at 10,000× g for 15 min. The total volume of supernatant was adjusted to 12 mL by methanol. Then 1 mL butanol was added, and the sample was taken to dryness using a Savant centrifugal evaporator. Methanol (2 mL) was added, and each sample was vortexed for 2 min. Samples were then passed through a 0.45 µm PTFE filter. The filter was washed with an additional 1.5 mL methanol. After adding 200 µL of 0.13 mg·mL^−1^ mangiferin (internal standard), the total volume was adjusted to 4 mL prior to analysis [25]. High-performance liquid chromatography-mass spectrometry (HPLC-MS) was used to separate and determine limonoid compounds described previously [26], and the column was Atlantis dC18 (2.0 × 100 mm, Waters, Medford, MA, USA). The main fragment ions, 471 m/z for limonin, and 515 m/z for nomilin were used for their quantification. Standards for identification were obtained from Hasegawa and coworkers [27,28].

### 2.5. Volatile Analysis

Juice samples in glass vials were crimp capped with Teflon/silicone septa. After incubation at 40 °C, direct-headspace (DHS) gas samples were analyzed by a gas chromatograph for the 2007 harvest [23], and a headspace-solid phase microextraction (HS-SPME) and GC-MS method was used for analysis of 2012 samples [29].

Standards were obtained from the following sources: acetaldehyde, hexanal, (Z)-3-hexenal, octanal, nonanal, decanal, (E,E)-2,4-decadienal, citral, β-sinensal, ethanol, hexanol, linalool, d-limonene, α-pinene, β-myrcene, γ-terpinene, nootkatone, ethyl butanoate, ethyl acetate, methyl butanoate, ethyl 2-methylbutanoate, and ethyl 3-hydroxyhexanoate were purchased from Sigma-Aldrich (Milwaukee, WI, USA), valencene was from Bedoukian (Danbury, CT, USA), and α-terpineol from Advanced Biotech (Totowa, NJ, USA). Quantification for each component was conducted by using a peak size *vs.* concentration curve built by serially diluted five point standard solutions [29]. Briefly, a standard compound was dissolved in pure methanol and the mixture was then introduced into a sugar equivalent deodorized orange juice (diluted “pumpout” concentrate) [11]. The range of concentrations in the standard curve for each compound covers the concentrations found in the samples. Other volatiles than listed here were not identified by comparing with the authentic chemical standards, but by comparing with library entries in NIST/EPA/NIH Mass Spectral Library 2011, as well as by comparing with published RIs [29].

### 2.6. Pectin and PME

Total pectin content was measured as galacturonic acid, determined using a microplate reader as described in Baldwin *et al.* [23]. Briefly, pectin was extracted from juice samples by adjusting to pH 2.4 and heating in a closed vessel reactor with microwave irradiation for 5 min. at 110 °C. After precipitation by using isopropyl alcohol, the extracts were hydrolyzed by pectinase (Pectinex Ultra SP-L, P-2611, Sigma-Aldrich, St. Louis, MO, USA) at pH 5 and 37 °C for 24 h. GA was determined by using anion exchange chromatography with a CarboPac PA1 column (Dionex Corp., Sunnyvale, CA, USA) and gradient elution using 0.0 to 0.5 M ammonium formate (Fluka #09735, Sigma-Aldrich, St. Louis, MO, USA) in water as mobile phase. For PME, 30 mL juice per sample was homogenized using a Brinkmann PT 10/35 homogenizer (Westbury, NY, USA) at speed 4 for 45 s. PME activity was determined titrimetrically with 0.5% citrus pectin [23].

### 2.7. Statistical Analysis

Analysis of variance (ANOVA) for each attribute/compound was conducted using the ANOVA procedure in SAS (Version 9.3; SAS Institute, Gary, NC, USA). Mean separation was determined by Tukey’s test at the 5% level. For multivariate statistical analyses, principal component analysis (PCA) and cluster analysis were performed using JMP (SAS Institute) to test the separation among harvest times based on the physical, chemical and biological measurements taken for this study. Heatmaps were generated based the average values by using Microsoft Excel 2013.

## 3. Results and Discussion

### 3.1. General Fruit and Juice Features

Average fruit weight increased by ~3% per month from February/March to April/May, and then decreased by ~4% in the last month in both 2007 and 2012 (Table 1), an occurrence associated with dehydration of the peel and browning of the fruit surface in the last month. Trends of peel oil content in the juice were similar to the fruit weight, increasing until April 2012, and then significantly decreasing in the last month, as much as 30% (Table 1). The maximum USDA designated oil content of “Grade A” OJ is 0.035% [30], while 0.01%–0.025% is preferred in most OJ [1]. Excessive peel oil in the OJ tends to be bitter or burning in taste character [31]. In this research, peel oil content in all juices were in the Grade A range with April juice having the highest content (Table 1). However, oil content in all the juice samples would have been much lower if the industrial juicer with the “Premium Setting” had been used, which is generally used to reduce peel oil content for “Valencia” fruit which have high levels of peel oil [1,23]. The increase in peel oil content before April 2012 may have been related to expanding oil glands during the development of peel tissues, and the decrease after April may have been due to the collapse of oil glands along with the dehydration of the peel [32]. Growth or regrowth of fruit and peel directly correlates to the enlargement of oil glands [31,33], and juice oils are overwhelmingly from these oil gland [12]. During senescence or under the stress of dehydration, cavities develop in oil glands of *Citrus* species, resulting in decreases of extractable peel oil yields [32]. The juice content to fruit weight decreased very slowly until mid-late season, and then substantially declined in the last month in both years (Table 1). The opposite trend was observed in insoluble solids content, which was unchanged until April 2012 and then sharply increased (Table 1). After reaching commercial harvest maturity, the fruit weight increased slowly in the early season, but in the later season the fruit underwent water loss from the peel, leading to reduced fruit weight and juice content, decreased turgor pressure in peel and collapsed oil glands. Thus, some of the peel oil could not be extracted, and with the decreased water content, the relative amount of insoluble solids naturally increased. Juice color number was high in the early season and decreased after mid-season (Table 1). Juice color is a primary quality attribute for orange juice. A USDA score of 36–40 is considered to be grade A, and 32–35 constitutes grade B for most citrus products [1]. A higher color number reflects deeper colored juices. Carotenoid pigments are responsible for orange juice color. Tropical climates generally grow fruit that produce lighter color juices [1].

**Table 1:** Changes of the basic fruit/juice properties, and non-volatile and mouth feel related attributes including fruit size, peel oil, insoluble solids, pectin, activity of pectin methylesterase (PME), juice color number, juice content, titratable acids (TA), citric acid, malic acid, soluble solids content (SSC), sucrose, glucose, fructose, SSC/TA ratio, limonin and nomilin in “Valencia” orange fruit/juice extracted from fruit harvested in March to June 2007 (*n* = 5) and February to May, 2012 (*n* = 4).

Attribute	Content/value in 2007				Content/value in 2012			
	Feb	Mar	Apr	May	Feb	Mar	Apr	May
fruit weight (g·fruit^−1^)	105 b ^a^	108 ab	111 a	107 b	144 b	148 ab	153 a	146 b
peel oil (g·100·g^−1^)					0.024 b	0.025 b	0.035 a	0.025 b
insoluble solids (g·100·g^−1^)					1.5 b	1.4 b	1.4 b	1.8 a
pectin (GA^b^, mg·g^−1^)	0.037 b	0.095 b	0.285 a	0.310 a	0.46 b	0.52 ab	0.53 ab	0.56 a
PME ^c^ activity (µmol·min^−1^·mL^−1^)					0.30	0.30	0.30	0.31
juice color number	39.5 a	39.7 a	38.5 b	38.8 b				
juice content (mL·100·g^−1^)	53 a	49 b	50 b	46 c	57 a	55 b	55 b	49 c
titratable acidity (TA, g·100·g^−1^)	0.82 a	0.68 b	0.57 c	0.43 d	1.07 a	0.93 b	0.74 c	0.68 d
citric acid (g·100·g^−1^)					1.06 a	0.80 b	0.62 c	0.62 c
malic acid (g·100·g^−1^)					0.02	0.02	0.02	0.02
soluble solids content (SSC, g·100·g^−1^)	10.7 ab	10.1 b	10.6 ab	11.0 a	12.1 c	12.7 b	13.2 ab	13.6 a
sucrose (g·100·g^−1^)	4.9 b	5.2 ab	5.5 a	5.6 a	4.8 c	5.1 bc	5.9 b	6.6 a
glucose (g·100·g^−1^)	1.9 a	1.9 a	2.0 a	2.0 a	3.7 ab	3.3 b	3.5 ab	4.0 a
fructose (g·100·g^−1^)	1.9 a	2.0 a	2.0 a	2.0 a	3.2 b	3.1 b	3.0 b	3.7 a
SSC/TA ratio	13.2 d	15.1 c	18.6 b	25.8 a	11 d	14 c	18 b	20 a
limonin (µg·g^−1^)	0.90 a	0.78 ab	0.67 bc	0.52 c	5.6 a	3.9 b	2.5 c	2.4 c
nomilin (µg·g^−1^)	0.22 ab	0.30 a	0.12 b	0.06 b	1.9 a	1.5 b	1.3 b	0.9 c

^a^ Values that are not followed by the same letter in the same row show significant difference at the 0.05 level using Tukey's test; ^b^ GA: galacturonic acid; ^c^ PME: pectin methylesterase.

### 3.2. Sugars and Acids

SSC of the juice in the 2007 harvest season did not follow a “gradual increase” pattern [3] because of the high value in February, which might be caused by nonuniform sampling. decreased very slightly then increased over the 2007 harvest season from April to June (Table 1) and was unusually low. It climbed steadily in 2012 to over 16% from February to May more in line with expectations (Table 1). Meanwhile, TA content decreased consistently over both harvest seasons as expected. Consequently, SSC/TA ratio increased steadily in both seasons to over 25 in June of 2007 and 20 in May 2012 (Table 1). All juices passed Florida juice standard (SSC/TA > 10.25), [3] however, high quality juice has a SSC/TA ratio between 12.5 and 19.5 [34]. The early harvested high acid fruit for both years had a SSC/TA ratio of around 11, out of the best quality range (Table 1). The last harvest in June of 2007 was too high (over 25, Table 1) to be in the optimal quality range due to an unpleasant overripe and insipid flavor that occurs when the SSC/TA ratio exceeds 20 [35,36]. Changes in TA and SSC/TA ratio are derived from some increase in SSC, but predominantly from a decrease in TA due to the decrease of citric acid, the dominant acid in citrus fruit. In mature orange juice sacs, both aconitase and citrate lyase activities were absent [37]. thus, decreasing the synthesis of oxaloacetate, the precursor of citrate, during maturation [38], which may play a major role in the acid decline over the season.

Individual sugars (sucrose, glucose and fructose) generally increased over the harvest seasons, but the significant differences only exhibited between March *vs.* May and June in sucrose in 2007, between first three months *vs.* the last month in fructose in 2012, between March *vs.* May in glucose in 2012, and among the different months in sucrose in 2012 (Table 1).

### 3.3. Limonoids

The bitter limonoids, limonin and nomilin generally decreased over the harvest season in both years, although the levels in 2012 were substantially higher than that in 2007 (Table 1). The results in this experiment showed that the fruit harvested in February 2012 had highest limonin levels, 5.6 µg·mL^−1^ (Table 1); it is close to the group threshold of 6 µg·mL^−1^ reported by Guadagni *et al.* [39]. The limonin content decreased gradually throughout the season (Table 1). Robertson and Nisperos [40] reported that limonin levels decreased in citrus fruits with advancing maturity. Kimball [41] pointed out that dilution and degradation during ripening causes a reduction in limonin levels. Dea *et al.* [42] reported that limonin and nomilin were shown to act synergistically, and together had a lower threshold than either compound alone [42].

Even though bitterness is considered to negatively impact OJ [42], a certain degree of bitterness is expected, and when combined with other desirable attributes, such as caffeine in coffee and alcohol in wine, bitterness can be an acquired taste in juice [43,44].

### 3.4. Pectin and PME Activity

Pectin, together with hemicellulose, cellulose and other minor components [45,46] make up the OJ cloud particles which contribute to the characteristic flavor, color and mouthfeel of the juice [11,25]. Galacturonic acid is the main component of pectin [47], thus total pectin was measured as galacturonic acid. Galacturonic acid content in juice increased from 0.037 mg·g^−1^ in March to 0.31 mg·g^−1^ in June in 2007, which is over an 8-fold increase. In 2012, pectin levels were generally consistent over the season, although there was an increase of about 20% from February to May, and juice from the later harvested fruit had wide variability (Table 1). The results are in contrast to Sinclair and Jolliffe [48] and Rouse *et al.* [49] who observed that in maturing oranges, total pectin and water-soluble pectic substances decreased in the peel and pulp, in both California and Florida “Valencia” fruit. However, Rouse and Moore [50] reported that pectin content in “Valencia” OJ increased in the 1973 season, but decreased in 1974. A possible reason for the increase of juice pectin is that when fruit were harvested later, a softening of albedo and membrane tissues may have occurred that can result in small amounts of these materials entering the juice during processing [50,51]. Because of the high pectin content in juice, the later harvested fruit can be problematic for potentially high cloud loss. PME is an enzyme that demethylates pectin in cell walls and can destabilize the cloud in OJ [23,52]. However, PME activity stayed stable over the season (Table 1).

### 3.5. Volatiles

**Figure 1:**
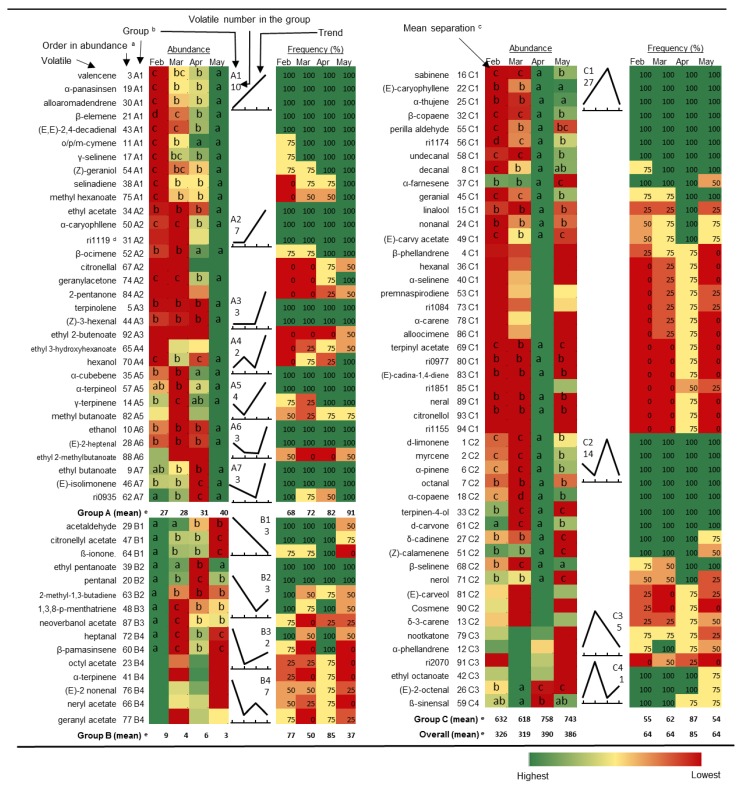
Changes in volatile abundance and frequency of detection in “Valencia” orange juice samples harvested from February to May 2012 (*n* = 4). ^a^ order in abundance listed by average peak size (total ion current) from high to low in total of 94 peaks; ^b^ groups: A-increase; B-decrease; C-peak with sub-groups in numbers; ^c^ the same letter within the same row (compound) represents no significant different at 0.05 level; ^d^ unknown compound with the retention indices (ri + a 4 digital number); ^e^ Average abundance (x 10^7^ total ion current) and frequency of detection (%).

In 2012, a total of 94 volatile compounds detected by the HS-SPME-GC-MS analysis are listed, where 32 compounds were detected in all samples, 31 in 50%—<100% of samples, and remaining 31 less than 50%, in which, 6 compounds, mostly terpenes were only detected at one harvest time, generally in April (Figure 1). The compounds were divided into three groups (A–C) by increasing and decreasing concentration patterns during the season (Figure 1). Group A consisted of 32 compounds that generally increased or changed little in the early months and for which the highest level was found in May (Figure 1). The representative chemical classes in this group are ethyl and methyl aliphatic esters, short chain (≤carbon 6) aldehydes, alcohols and ketones, and sesquiterpene hydrocarbons, such as the important and/or abundant OJ aroma contributors ethyl butanoate, ethyl 2-methylbutanoate and ethyl acetate, (Z)-3-hexenal, ethanol and hexanol, and valencene (Table 2). There were 7 sub-groups (A1–A7) in this group. Ten compounds in A1 continually increased, and another 10 compounds in A2 and A3 remained low for the first one or two months and then increased (Figure 1). The other 4 sub-groups (A4–A7) containing 12 compounds, all reached their highest levels in the last month, however, fluctuated in the early stages (Figure 1). The average detection frequency in this group was 68% in February, and continually increased to 72%, 82% and 91%, in following months, which follow the same trend as for the average peak sizes, although there were 16 compounds that were detected in all stages in this group (Figure 1). It is generally recognized that the sesquiterpene hydrocarbons are derived from acetyl-CoA through the mevalonic acid (MVA) pathway in the cytosol [53,54]. Valencene, the dominant sesquiterpene hydrocarbon, occurs in both peel oil (from oil glands in the flavedo) and juice oil (from oil bodies in the juice sacs). Since the OJ essence oil contains a high level of valencene, juice oil is recognized as a major source [12].

Ethanol and ethyl moieties from esters are derived from the fermentation process, and the increased fermentation that occurs with progressed maturity is most likely the cause of enhanced production of those chemical classes [55,56]. The accumulation of methanol in ripe tomato fruit was likely the source of generated methyl esters with increased PME activity [57]. Beekwilder *et al.* [58] confirmed that the availability of alcohol substrates is an important parameter to consider when engineering volatile ester formation in plants. Carbon 5 and 6 aldehydes and alcohols are derived from polyunsaturated fatty acids, via the lipoxygenase (LOX) pathway [59], and the accumulation in the late stages of ripening could be accelerated by hydrolysis of membrane lipids during the maturity progresses [60,61]. For ethyl 2-methylbutanoate, which contains branch-chained acid moieties derived from isoleucine [62], the increased production with progressed maturity, may mostly be due to increased availability of ethanol, rather than the biosynthesis of the acid moieties [56].

Group B consisted of 15 components which were at highest level in February, and decreased thereafter (3 components in B1 sub-group) or fluctuated (12 components in B2-B4 sub-groups) in the later months (Figure 1). The average peak areas in this group were 9, 4, 6 and 3 (× 10^6^), from February to May (Figure 1). The average detection frequency was 77% in February, and decreased to 50% and 37% in March and May, respectively (Figure 1). However, the value was 83% in April, higher than any other months (Figure 1). This group included many esters, and the representative chemicals were acetate esters, including four terpenic and one aliphatic acetate. It seems that the large increase in ethanol at later stages outcompeted the other alcohol moieties to be esterified [56]. Acetaldehyde increased in reduction capacity in the fermentation pathway. Beltrán, *et al.* [63] observed the accumulation of both ethanol and acetaldehyde in olive fruit during maturation, although ethanol showed a more rapid increase.

Group C consisted of 47 components, 50% of total volatile compounds detected in this research, which peaked in March or April (Figure 1). The representative chemical classes in this group included monoterpene hydrocarbons and straight-chain saturated aldehydes (C8–C10). Most of these compounds, 41 in total, peaked in April, which coincided with the change in peel oil content (Table 1 and Figure 1). The average detection frequency followed the same trend as for the change of concentration: 55%, 62%, 87% and 54% in February to May (Figure 1). Monoterpene hydrocarbons are derived from pyruvic acid, and biosynthesized through the 2-C-methyl-d-erythritol-4-phosphate (MEP) pathway in plastids [64]. In orange juice, monoterpenes are predominantly from the peel oil and introduced into juice through processing [9,12,23,25]. Chemicals in this class comprise over 90% of total citrus volatiles and play a fundamental role in citrus aroma [12,29]. Many reports showed that the homologous straight-chain aldehydes, octanal, nonanal and decanal, are associated with peel oils [12]. Together with other aldehydes, this class of chemicals play a major role in orange flavor. Total aldehyde content has been used as one of the industry standards for high quality cold-pressed peel oil [65], although not all aldehydes contribute positive flavor to orange juice [12]. The decease of peel oil content, along with the majority of volatiles associated with peel oil after April coincided with fruit (especially peel) dehydration, perhaps was caused by the collapsing of oil glands [66].

Table 2 listed 23 important aroma contributors in orange juice [10,11,67] and their changes during the 2007 and 2012 harvest seasons. In 2007, DHS sampling method was used and only 22 volatiles were detected, and of which 13 important compounds were listed in Table 2. Overall, d-limonene, myrcene, linalool, β-sinensal, acetaldehyde, hexanal, (Z)-3-hexenal, octanal, decanal, (E,E)-2, 4-decadienal, ethyl butanoate, and ethyl 2-methyl butanoate were present at or above their odor threshold, *i.e.*, the odor active value (OAV) > 1 for at least one harvest (Table 2). It is important to note that the odor thresholds listed are based on an OJ matrix, and the values are much higher than those reported for water based thresholds [10,11].

**Table 2:** Change of 23 important comtributers to orange juice aroma quality in juices extracted from “Valencia” oranges harvested from March to June 2007 and Feburary to May 2012.

Compound	Oder Description ^a^	Threshold in OJ Matrix (µg·mL^−1^) ^b^	Concentration (µg·mL^−1^) by DHS in 2007									Concentration (µg·mL^−1^) by HS-SPME in 2012								
			Group ^c^	Mar		Apr		May		Jun		Group ^c^	Feb		Mar		Apr		May	
d-limonene	Citrus, lemon, minty	13.3	C1	58	b ^d^	90	b	336	a	302	a	C2	224	c	217	c	298	a	248	b
myrcene	Mossy, musty, geranium	0.5	C1	0	b	0	b	2.64	a	2.53	a	C2	0.86	c	0.78	c	1.42	a	1.15	b
α-pinene	Resin, pine tree, ethereal	2.0	C1	0.1	b	0.15	b	0.83	a	0.79	a	C2	0.38	c	0.32	c	0.66	a	0.53	b
γ-terpinene	Sweet, citrus	2.1 ^e^										A5	0.0038	b	0.0010	c	0.0049	ab	0.0060	a
valencene	Lemon, floral	10.5	B2	2.71	a	2.59	a	2.46	a	2.49	a	A1	2.1	c	2.5	bc	2.8	b	3.3	a
linalool	Floral, fruity, sweet	0.1	C1	1.66	c	2.1	b	3.15	a	2.9	a	C1	0.17	b	0.12	b	1.05	a	0.20	b
α-terpineol	Lemon, minty, piney	9.1	A7	0.94	a	0.8	b	0.76	b	0.99	a	A5	0.15	ab	0.14	b	0.17	a	0.20	a
nootkatone	Grapefruit, green	3.1										C3	0.080		0.12		0.11		0.03	
geranial	Citrus, minty, green	0.7										C1	0.031	c	0.022	c	0.042	a	0.036	b
neral	Citrus, lemon, minty	0.7										C1	tr	b	tr	b	0.0033	a	tr	b
β-sinensal	orange, fruity	0.004 ^f^										C4	0.0023	c	0.0030	c	0.0083	a	0.0061	b
acetaldehyde	Fresh, fruity, solvent	0.3	C4	4.9	a	8.1	a	7.2	a	7.8	a	B1	4.1	a	3.7	a	2.2	b	1.6	b
hexanal	Grassy, green, soapy	0.09	A5	0.04	b	0.02	c	0.04	b	0.07	a	C1	0.000		0.03		0.27		0.00	
(*Z*)-3-hexenal	Green, grassy	0.02										A3	0.019	b	0.017	b	0.016	b	0.047	a
octanal	Floral, citrus, green	0.1	C1	0	b	0.04	b	0.53	a	0.05	b	C2	0.12	b	0.10	b	0.21	a	0.12	b
nonanal	Citrus, floral, soapy	0.2										C1	0.004	b	0.006	b	0.017	a	0.006	b
decanal	Citrus, fatty, green	0.1	C1	0.05	c	0.05	c	0.26	a	0.01	b	C1	0.143	c	0.180	b	0.287	a	0.257	ab
(E,E)*-2,4*-decadienal	Fatty, waxy, green	0.004										A1	0.027	b	0.033	c	0.061	b	0.079	a
ethyl acetate	Fruity, solvent	11.0	B4	3.86	a	3.27	b	3.43	b	3.02	c	A2	0.24	b	0.25	b	0.26	b	0.49	a
ethyl butanoate	Fruity, pineapple	0.005	C3	0.06	b	0.86	a	0.09	a	0.08	a	A7	0.90	ab	0.89	b	0.77	b	1.21	a
methyl butanoate	Fruity, stawberry	0.4	C1	0.01	b	0.02	ab	0.04	a	0.03	ab	A5	0.014		0.007		0.019		0.025	
ethyl 2-methylbutanoate	Fruity	0.0002										A6	0.025		tr		tr		0.038	
ethyl 3-hydroxyhexanoate	Citrus	10.2										A4	0.325		0.155		0.183		0.197	

^a^ Descripters were from Perez-Cacho and Rouseff [9], Plotto *et. al*. [10] and Qiao *et al*. [68]; ^b^ Retronasal identification threshhold in a reconstituted pump-out adapted from Plotto *et al*. [10,11] except described otherwise; ^c^ Group: A: increased; B: decreased; C: peaked. See Figure 1 for details; ^d^ Values followed by different letters in the same compounds and same year (row) are significant different at *p* = 0.05 using Tukey’s test; ^e^ Threshold in water [11]; ^f^ Threshold in water [69].

**Figure 2:**
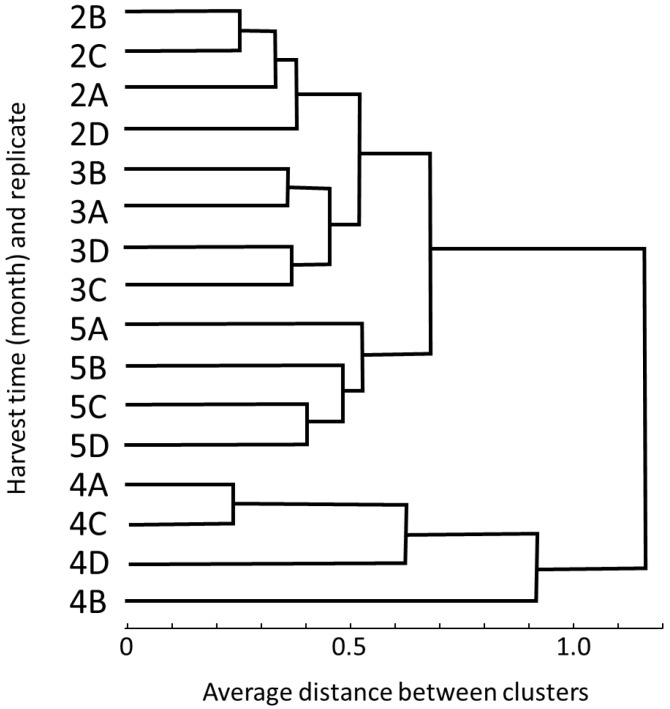
Cluster analysis of aroma quality of “Valencia” orange juice harvested from February to May 2012 based on 94 volatile compounds. “2”–“5” represent months (2 = February, 3 = March, 4 = April and 5 = May) and A–D represent replicates.

Cluster analysis (Figure 2) using all 94 volatile components showed that all samples discriminated by their harvest time, with February and March samples being more closely associated each other and April samples having the most distance from the other harvests (Figure 2). 

More than half of the important volatiles, 9 out of 13 compounds in 2007, and 12 out of 23 compounds in 2012 were in group C, and generally peaked in mid-late seasons (Table 2). The typical chemical classes were monoterpenes, straight-chain aliphatic and terpenic aldehydes (Table 2). The most abundant volatiles in OJ are monoterpenes, especially d-limonene, which counts for about 95% of total peel oil and is an important contributor to citrus and orange aroma [12,68]. Another two abundant and important monoterpene aroma contributors are α-pinene and myrcene. The firmer makes a positive contribution to orange flavor and the latter can be a negative contributor [68,69], denoting pungency and bitterness at high concentration [70]. All aroma active terpenic and non-terpenic aldehydes determined in this research were in group C except acetaldehyde, (Z)-3-hexenal and (E,E)-2,4-decadienal. In the group C, neral and geranial are monoterpenic aldehydes, and possess a lemon, citrus-like odor, and β-sinensal, a sesquiterpenic aldehyde, possesses a sweet aroma (Table 2). α-Sinensal was not detectable in this experiment as the α- isomer concentration is only half that of the β-sinensal in OJ [71]. The odor properties of octanal, decanal and nonanal are slightly different from each other. All of them are pungent, with octanal and nonanal producing a citrus-like odor and decanal eliciting a cilantro note [72]. Hexanal contributes a green and grassy aroma, but may not be an important flavor attributor to OJ [9]. Linalool is generally from peel oil and a major odorant in commercial juice, contributing a sweet floral odor [73]. Nootkatone is a sesquiterpene ketone and characterized as having a grapefruit flavor [12]. However, it may contribute a citrus-like background to orange juice [9]. In comparison to group C, the increase in concentration of group A chemicals did not stop after April, and reached a maximum in May (Figure 1). Firstly, the five ethyl esters all increased continually in 2012, but reached peak or flatted out before the last harvest in 2007. Ethyl butanoate, with a high concentration and low threshold, is one of the single most important aromas to OJ with a fruity top-note [9]. Valencene, a sesquiterpene hydrocarbon, has little direct effect on OJ flavor due to its high odor threshold [12,68], but is very abundant, and like limonene can affect headspace partitioning and, thus, the perception of other volatiles [10]. In hand-squeezed fresh OJ, with low peel oil content, valencence played an important role in contributing a citrus-like aroma note [74]. α-Terpineol, formed from d-limonene and linalool, may contribute either a positive (floral, lilac-like) or negative (turpentine-like, musty, pungent) role in fruits, however, it is often used as an indicator for canned juice that has been stored too long [75]. Reasons for the continual increase of this compound together with γ-terpinene, (Z)-3-hexenal and (E,E)-2, 4-decadienal are unknown. (Z)-3-Hexenal has a lower threshold than hexanal and contributes a green top-note to fresh OJ [9]. (E,E)-2,4-Decadienal in citrus oils is produced by oxidative breakdown of the long chain fatty acids present in the oils [9,76], and provides a positive aroma contribution to orange juice [76]. γ-Terpinene contributes a sweet and citrus aroma [9]. There were two compounds, valencene and ethyl acetate in 2007, and one compound, acetaldehyde in 2012 in group B and decreased during the harvest season (Table 2). Acetaldehyde is considered important to orange flavor [12,36,77], and contributes a fresh fruity flavor [12]. As discussed earlier, both valencene and ethyl acetate have little aroma impact due to their high odor thresholds. 

### 3.6. Overall Flavor Quality

**Figure 3:**
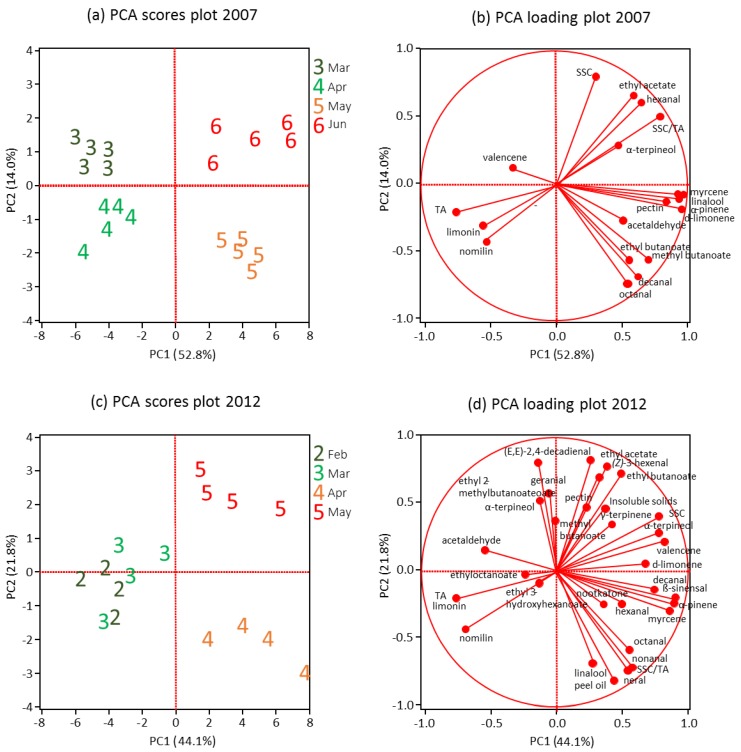
Principle component analysis (PCA) of important attributes of aroma, taste, and mouth feel components of Valencia’ orange juice harvested over the 2007 and 2012 seasons. (**a**) and (**b**) PCA scores plot and loading plot in 2007, respectively. (**c**) and (**d**) PCA scores plot and loading plot in 2012, respectively. Component numbers for volatile, non-volatile and mouth feel attributes were 13, 5 and 1, respectively in 2007 and 23, 5, and 3, respectively in 2012.

To get a clear picture of the changes in flavor chemicals over the harvest season, PCA analysis were performed by using the major orange juice taste contributors for sweetness, sourness and bitterness (SSC, TA, SSC/TA, limonin and nomilin), as well as other components that can indirectly influence OJ flavor and mouthfeel (peel oil, pectin and insoluble solids) and the 23 important aroma volatile compounds listed in Table 2 for 2012 samples (Figure 3c,d). There were less aroma compounds and mouthfeel attributes detected in the 2007 samples, although similar PCA procedure was performed (Figure 3a,b). For the 2007 samples, the PCA components 1 and 2 (PC1 and PC2) explained 52.8% and 14.0% of the variation, respectively (Figure 3). The score plot (Figure 3a) shows a clear separation of early (March and April) and late (May and June) harvested samples by PC1, and the sub-clusters by PC2 (Figure 3a). The factor loadings plot (Figure 3b) shows that TA, limonin, nomilin, and valencene were associated with early harvested OJ, although valencene played only a small role (Figure 3b). Late harvested, May and June 2007 fruit were associated with d-limonene, α-pinene, myrcene, linalool and pectin. May fruits more related to octanal, decanal, ethyl butanoate and methyl butanoate, while June fruits more related to SSC, SSC/CA ratio, ethyl acetate, and hexanal (Figure 3b). Acetaldehyde and α-terpineol played little role (Figure 3b).

The 2012 data exhibited similar patterns to the 2007 data (Figure 3). The PC1 and PC2 explained 44.1% and 21.8% of the variation, respectively (Figure 3c and 3d). The score plot (Figure 3c) shows a clear separation of early (February and March) and late (April and May) harvested samples by PC1, and April and May samples by PC2 (Figure 3a). February and March samples were not separated clearly, although more samples in March were closer to the later harvested fruit by PC1 (Figure 3a). The factor loadings plot (Figure 3b) shows that TA, limonin, nomilin, ethyl butanoate, ethyl acetate, (Z)-3-hexenal, SSC, valencene, α-terpineol, sinensal, α-pinene, myrcene, octanal, nonanal, neral, and peel oil exhibited strong discrimination power, ethyl 3-hydroxyhexanoate, ethyl octanoate, methyl butanoate, hexanal, and nootkatone played only small roles in discriminating samples, and others were in between in contributing to the discrimination of samples. Small conflicts between the two years were seen in some volatile compounds, such as valencene and acetaldehyde, but they played little roles in the discrimination (Figure 3b,d).

**Figure 4:**
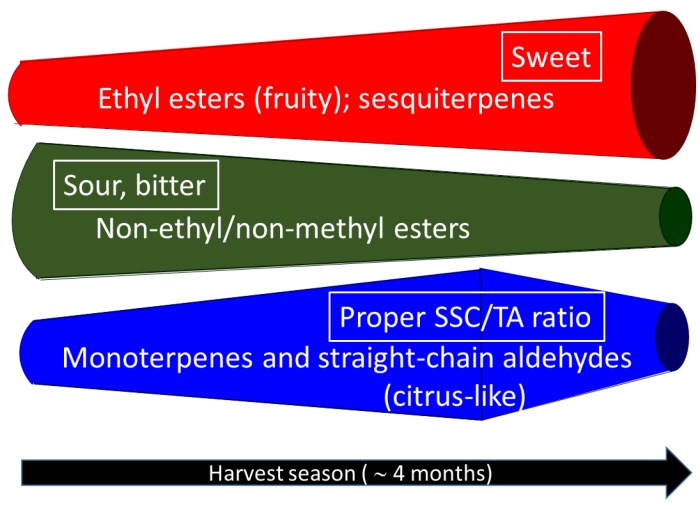
A schematic diagram to demonstrate the change patterns of different non-volatile (boxed) and volatile (non-boxed) flavor chemical compounds along with harvest time (from February/March to May/June) in “Valencia” orange juice. The width of the band indicates the relative abundance of each chemical class/attribute except for SSC/TA ratio which represents the optimal quality.

There are many indications that aroma chemicals (volatiles), taste chemicals and the matrix interact physically and chemically and in terms of flavor perception [11,78]. Nonanal, octanal and decanal in all samples were found at subthreshold concentrations (Table 2), thus, their aromas should not be detectable individually, but may enhance each other in combination [78]. Similar effects have been found with ethyl esters, and monoterpenes in OJ [9,12]. Esters with fruity aroma often enhance fruit sweetness and suppress sourness [78]. Futhermore, the juice matrix in OJ influences complicates flavor perception. Brat, Rega, Alter, Reynes and Brillouet [15] and Radford, Kawashima, Friedel, Pope and Gianturco [16] showed that terpene hydrocarbons were associated with OJ pulp and ethyl butanoate and octanal were mainly in aqueous solution, indicating the influence of increased insoluble solids and pectin content on flavor perception later in the season due to changes in headspace partitioning of some compounds like terpene hydrocarbons (Figure 4).

## 4. Conclusions

“Valencia” oranges harvested in the middle to late season (May 2007 and April 2012) are preferred for high quality juice with optimum SSC, TA, and SSC/TA ratio, and better volatile profiles. The early harvested fruit had high juice yield, but also had undesirable levels of bitter and sour compounds and lower levels of volatiles. After reaching optimal quality, monoterpene compounds, representing citrus-like aromas, decreased along with peel oil, although ethyl esters with the fruity top-note increased continually. With the SSC/TA ratio approaching 20 in later harvested samples, the juice quality falls out of the set commercial parameters for orange juice. In total, 94 volatiles were detected in the 2012 juice samples, and separated to 3 groups by the increase/decrease trends during the harvest season: 32 components in group A increased continually, including important and/or abundant OJ aroma contributors, such as ethyl butanoate, ethyl 2-methylbutanoate, ethyl acetate, (Z)-3-hexenal, ethanol, hexanol, and valencene; 15 components in group B decreased in general, including acetaldehyde and some acetate esters; rest of 47 components, as group C, including d-limonene, myrcene, α-pinene, octanal, nonanal, and sinensal, mostly peaked at April and some at March. The average detected peak number in April samples were 85, in comparison with 64 in February, March and May harvested samples.

## 5. Disclaimer

This article is a US Government work and is in the public domain in the USA. Mention of a trademark or proprietary product is for identification only and does not imply a guarantee or warranty of the product by the US Department of Agriculture. The US Department of Agriculture prohibits discrimination in all its programs and activities on the basis of race, color, national origin, gender, religion, age, disability, political beliefs, sexual orientation, and marital or family status.
